# A qualitative stakeholder analysis of beliefs, facilitators, and barriers for a feasible prehabilitation program before lung cancer surgery

**DOI:** 10.1007/s00432-023-05298-6

**Published:** 2023-09-05

**Authors:** M. J. J. Voorn, E. M. W. Bastiaansen, C. D. Schröder, V. E. M. van Kampen-van den Boogaart, G. P. Bootsma, B. C. Bongers, M. L. G. Janssen-Heijnen

**Affiliations:** 1 Department of Clinical Epidemiology, VieCuri Medical Center, Tegelseweg 210, 5912 BL Venlo, The Netherlands; 2 Adelante Rehabilitation Center, Venlo, The Netherlands; 3 Department of Epidemiology, GROW School for Oncology and Reproduction, Faculty of Health, Medicine and Life Sciences, Maastricht University, Maastricht, The Netherlands; 4 Physical Therapy Practice, Tante Louise, Bergen op Zoom, The Netherlands; 5 Ecare4you, Amersfoort, The Netherlands; 6 Department of Pulmonology, VieCuri Medical Center, Venlo, The Netherlands; 7 Department of Pulmonology, Zuyderland Medical Center, Heerlen, The Netherlands; 8 Department of Nutrition and Movement Sciences, Nutrition and Translational Research in Metabolism (NUTRIM), Faculty of Health, Medicine and Life Sciences, Maastricht University, Maastricht, The Netherlands; 9 Department of Surgery, Nutrition and Translational Research in Metabolism (NUTRIM), Faculty of Health, Medicine and Life Sciences, Maastricht University, Maastricht, The Netherlands

**Keywords:** Non-small cell lung cancer, Preoperative care, Preferences, Patient experience, Qualitative research, surgery

## Abstract

**Background:**

In order to develop a feasible prehabilitation program before surgery of NSCLC, this study aimed to gain insight into beliefs, facilitators, and barriers of (1) healthcare professionals to refer patients to a prehabilitation program, (2) patients to participate in and adhere to a prehabilitation program, and (3) informal caregivers to support their loved ones.

**Methods:**

Semi-structured interviews were conducted with healthcare professionals, patients who underwent surgery for NSCLC, and their informal caregivers. The capability, opportunity, and motivation for behavior-model (COM-B) guided the development of the interview questions. Results were analyzed thematically.

**Results:**

The interviews were conducted with twelve healthcare professionals, seventeen patients, and sixteen informal caregivers. Four main themes were identified: (1) content of prehabilitation and referral, (2) organizational factors, (3) personal factors for participation, and (4) environmental factors. Healthcare professionals mentioned that multiple professionals should facilitate the referral of patients to prehabilitation within primary and secondary healthcare involved in prehabilitation, considering the short preoperative period. Patients did not know that a better preoperative physical fitness and nutritional status would make a difference in the risk of postoperative complications. Patients indicated that they want to receive information about the aim and possibilities of prehabilitation. Most patients preferred a group-based physical exercise training program organized in their living context in primary care. Informal caregivers could support their loved one when prehabilitation takes place by doing exercises together.

**Conclusion:**

A prehabilitation program should be started as soon as possible after the diagnosis of lung cancer. Receiving information about the purpose and effects of prehabilitation in a consult with a physician seems crucial to patients and informal caregivers to be involved in prehabilitation. Support of loved ones in the patient’s own living context is essential for adherence to a prehabilitation program.

## Introduction

Lung cancer has increased significantly in recent decades, contributing to approximately 13% of all cancer diagnoses worldwide (World Cancer Research Fund International 2020) Non-small cell lung cancer (NSCLC) constitutes the majority (85%) of lung cancers (Netherlands Cancer Registry 2020). The primary curative treatment for patients with early-stage NSCLC is surgical tumor resection (Senan 2013; Dong et al. 2019; Verstegen et al. 2013). Despite advances in surgery, such as video-assisted thoracic surgery, the incidence of postoperative complications remains high and occurs in 35% of patients with NSCLC (Dutch Lung Cancer Audit (DLCA) 2018). Research has shown that the risk for postoperative complications is higher in patients over 70 years with a low expiratory volume in one second (FEV_1_), a poor preoperative aerobic fitness, tobacco-related comorbidity, cognitive impairment, and/or comorbidities (e.g., chronic obstructive pulmonary diseases, cardiovascular diseases, and/or diabetes mellitus) (Kozower et al. 2010; Sebio Garcia et al. 2016; Leduc et al. 2017). Postoperative complications are associated with a delayed or incomplete recovery of physical fitness levels after surgery (Licker et al. 2017).

A multimodal prehabilitation program, including aerobic, resistance, and/or inspiratory muscle training, nutritional advice, and/or support for smoking-cessation, can reduce the risk of postoperative complications after surgery in patients with NSCLC (Sebio Garcia et al. 2016; Templeton and Greenhalgh 2019; Liu et al. 2019). Moreover, prehabilitation can decrease the length of hospital stay and facilitate postoperative recovery (Liu et al. 2019; Gravier et al. 2021; Granger and Cavalheri 2022). Despite the effectiveness of prehabilitation, it is not yet part of usual care. Previous studies in patients with NSCLC have shown that the ability to participate in a prehabilitation program is low (between 28 and 56% (Sebio Garcia et al. 2017)) and that program adherence is only moderate (between 53 and 73% (Granger et al. 2018)). In addition, to improve participation and adherence in prehabilitation, it is important to gain insight into preferences and possible facilitators and barriers of a prehabilitation program among patients, their informal caregivers, and healthcare professionals.

Surgeons see benefits of prehabilitation in order to decrease the risk of postoperative complications in patients with NSCLC and are willing to delay surgery with two weeks; however, it is unclear for surgeons when and where to refer to for prehabilitation (Shukla et al. 2020). Research in patients with colorectal cancer has shown that, next to ensuring a therapeutically valid program content, it is important to identify the barriers and preferences of patients in order to develop a feasible and (cost-)effective prehabilitation program in the proper context (Agasi-Idenburg et al. 2020; Thomas et al. 2019). Therefore, this study aimed to gain insight into beliefs, facilitators, and barriers of 1) healthcare professionals to refer patients to a prehabilitation program, 2) patients with NSCLC to participate in and adhere to a prehabilitation program, and 3) informal caregivers to support their loved ones in prehabilitation.

## Methods

### Study design

A qualitative interview study was performed to develop a feasible prehabilitation program, including physical exercise training, nutritional and psychological support, and/or coaching towards lifestyle changes for patients with operable NSCLC. Healthcare professionals, patients, and informal caregivers were interviewed to explore beliefs, facilitators, and barriers to prehabilitation. Included patients did not perform prehabilitation but had the experience of a perioperative period and could reflect on facilitating factors and barriers. In order to gain a respectable representation of beliefs, no exclusion criteria were set. This study was approved by the Medical Research Ethics Committee Zuyderland (reference number: 2021–2879). All participants were recruited between September 2021 and February 2022.

### Study population

#### Healthcare professionals

Healthcare professionals of disciplines involved in the treatment of patients with NSCLC with experience in prehabilitation of patients with cancer varied from pulmonologists, rehabilitation physicians, pulmonary nurses, psychologists, dieticians, and physical therapists. Names of these specialists were provided by the researcher (MV) of VieCuri Medical Center, Venlo, The Netherlands, and a pulmonologist (GB) of the Zuyderland Medical Center, Heerlen, The Netherlands.

Healthcare professionals were informed and invited to participate in the study by e-mail by the researcher. After e-mail consent, the researcher contacted the included healthcare professionals to schedule an interview. Written informed consent was provided at the start of the interview.

#### Patients with NSCLC who underwent lung resection

Potentially eligible patients were identified in the multidisciplinary team meeting in the VieCuri Medical Center or Zuyderland Medical Center by the researcher (MV), case manager (nurse specialist in lung oncology) by screening surgery schedules. Eligibility criteria were (1) patients who underwent lung resection for NSCLC, (2) ≥ 18 years of age, (3) adequate understanding of the Dutch language, and (4) able to participate within 30 days after surgery. The pulmonologist provided information regarding the study during the first consultation after hospital discharge following lung resection. Interested patients received a patient information letter. Thereafter, the researcher contacted the patient to verify the willingness to participate and to schedule an interview after oral consent. Written informed consent was obtained before interviewing.

#### Informal caregivers of patients with NSCLC

During the first consultation after discharge from the hospital following lung resection, interested patients were asked to identify an informal caregiver who had been vital to them in the perioperative period. This could be a spouse, an adult child, a close friend, or a relative. The researcher contacted the informal caregiver by phone to ask for oral consent to schedule an interview. Written informed consent was signed at the start of the interview.

### Data collection

Data was collected through one-to-one semi-structured interviews at a time and place suited for each participant. Interviews with healthcare professionals were conducted via video consulting online. For patients and informal caregivers this was at their home or before or after a scheduled usual care appointment at the hospital. One researcher (MV) conducted the interviews with patients and informal caregivers separately. The other researcher (EB) conducted the interviews with healthcare professionals. The number of interviews intended to perform was based on inductive thematic saturation. Saturation was considered when interviews did not lead to new themes. It was expected that approximately ten interviews with healthcare professionals, fifteen interviews with patients, and fifteen with informal caregivers were required. Preoperative and postoperative patient characteristics were derived from the electronic patient files. When applicable, a patient’s postoperative complications were graded according to the Clavien-Dindo classification (Clavien et al. 2009) to provide insight into treatment characteristics and treatment outcomes of the patients.

### Content of the interviews

The interviews were conducted based on a semi-structured interview guide (developed by MV, CS, BB, and MJ) using open-ended questions that initially defined the areas explored. Interview topics are shown in Table 1. These initial topics were chosen based on an existing behavior model. The capability, opportunity, motivation, and behavior (COM-B) model (Michie et al. 2011) guided the categories of questions regarding three types of behavior: (1) participating in multimodal prehabilitation, (2) referring to a prehab program, or (3) to support a loved one during prehab. The COM-B model (Michie et al. 2011) suggests that engagement in a behavior is determined by capability (e.g., physical skills, knowledge), opportunity (e.g., environment, social norms), and motivation (e.g., habits, beliefs, general attitude towards multimodal prehabilitation). Three test interviews were conducted for the study population, after which the topics and interview guides were optimized. The researchers (MV and EB) discussed changes in the interview guides.

**Table 1:** Interview topic guide

1A. Healthcare professionals	
Behavior	1. Do you refer patients for prehabilitation, physical therapy, nutritional support, smoking cessation? To whom/what most? 2. Do you think patients would participate in prehabilitation? 3. Which element of prehabilitation is the most important for your patients?
Motivation	4. Do you think it makes sense to offer prehabilitation to your patients? 5. In your opinion, is aerobic fitness related to the development of complications and recovery after surgery? 6. For which group of patients do you think referral to prehabilitation would be useful/not useful? 7. Are you planning to refer your patients with operable non-small cell lung cancer to prehabilitation? 8. How often do patients suffer from complications after lung surgery?
Capability	9. Would it be difficult or easy for you to estimate whether someone qualifies for prehabilitation? 10. Do you think all operable patients with NSCLC are eligible? 11. What are barriers for you to refer patients to prehabilitation? 12. What would make it easier for you to refer patients for prehabilitation?
Opportunity	13. Do you think it is logistically feasible to set up multimodal prehabilitation for patients preparing for lung cancer surgery, in combination with the appointments that patients have regarding diagnostics and treatment? 14. Do you know what the procedure is to refer patients for prehabilitation? Is this difficult or easy to figure out and implement?
Other questions	15. Do you think that preparing patients for surgery is needed by means of prehabilitation? 16. Which healthcare providers could best guide the patient in the preoperative period? 17. What are your thoughts about a lifestyle clinic in the hospital? 18. What are your thoughts about professional guidance for patients in preparation of surgery? 19. What are your thoughts about extending a delay before surgery to make more time for prehabilitation? How long might this delay be?
1B. Patients	
Behavior	1. How did you experience the period around your surgery? 2. Did you do anything specific in preparation for your surgery in the period prior to your surgery (e.g., physical exercise training, nutritional adjustments)? 3. How do you look back on this period? Would you do anything else with today’s knowledge? 4. Did your physician advise you to be physically active/perform physical exercise training, adjust your diet, and/or stop smoking in preparation of your surgery? 5. Have you heard of prehabilitation? What are your thoughts about such a program?
Capability	6. Were you able to perform physical exercise training before your surgery? Did you do this? 7. Do you think you were able to follow a prehabilitation program at least 3 times a week? 8. Do you think you were able to follow a protein-rich diet? *For smokers:* 9. Did you stop smoking before surgery? Did you consider stopping? 10. Do you think you were able to stop smoking?
Motivation	11. Do you think it makes sense to perform physical exercise training prior to your surgery? 12. Do you think it is useful (for you) to eat a protein-rich diet and to adjust your eating behavior prior to your surgery? 13. Do you think it makes sense (for you) to get support from a psychologist prior to your surgery? 14. If you had to do it all over, how would you estimate the chance that you would follow a physical exercise training program in preparation of your surgery? *For smokers:* 15. If applicable: do you think it would be beneficial (for you) to quit smoking prior to your surgery?
Opportunity	16. Were you able to perform physical exercise training before your surgery? 17. Were you able/possible to follow a protein-rich diet before your surgery? 18. Did you have enough time to perform physical exercise training before your surgery? *For smokers:* 19. Were you able to quit smoking before the surgery?
Other questions	20. Did you have information before the operation about physical exercise training, nutrition, smoking cessation, psychological counseling, prehabilitation programs? 21. How physically fit did you feel before surgery after being diagnosed with lung cancer? 22. How physically fit did you feel after surgery? 23. Have you had any complications? 24. What do you think about guidance from a healthcare provider about prehabilitation programs? 25. What if the preoperative time period before surgery was extended in order to be able to participate in prehabilitation to be better prepared?
1C. Informal caregivers	
Behavior	1. How are you? How did your loved one experience the period around his/her surgery? 2. Did you and your loved one do anything specific in preparation for surgery? 3. Have you ever heard of prehabilitation? What do you think about that? Do you think it would make sense for your loved one? 4. Has your loved one been offered prehabilitation or rehabilitation? 5. Have you assisted your loved one in a prehabilitation program/or would you have been able to assist your loved one if he/she had been offered this before the surgery? 6. Did you need support during this period?
Motivation	7. Do you think it is useful (for your loved one) to perform physical exercise training prior to the surgery? Did you support your loved one to become more physically active or participate in physical exercise training? How did you do that? Do you think there is a role for the informal caregiver in a patient’s preparation for surgery? 8. Do you think it is useful (for your loved one) to follow a protein-rich diet before surgery? Did you support your loved one to eat differently? Do you see a role for yourself here? *For informal caregivers of patients who smoke:* 9. Do you think it was useful (for your loved one) to stop smoking before his/her surgery? Do you see a role for yourself here? Did you help your loved one to stop smoking? If so, how? 10. Do you think it is useful (for your loved one) to receive support from a psychologist prior to the surgery? Do you see a role for yourself here?
Capability	11. Do you feel you are able to support your loved onein the preparation for surgery in terms of physical exercise training and dietary adjustments? *For informal caregivers of patients who smoke:* 12. Do you feel able to support your loved one to quit smoking? 13. Were you able to help your loved one to stop smoking before the operation?
Opportunity	14. Did you have enough opportunities (e.g., time) to support your loved one in his/her preparation for surgery? 15. Could you change something in the environment to make it easier for your loved one to be more physically active?

Healthcare professionals were asked about the facilitators and barriers concerning their ability and available opportunities to refer patients to prehabilitation. Additionally, their opinion about which subgroup(s) of patients benefits the most from prehabilitation was asked. Information requested included patient characteristics, previous experiences with physical exercise training, nutritional advice, and smoking-cessation. Moreover, the occurrence of postoperative complications and their opinion about surgical delay to gain time for prehabilitation were questioned. Informal caregivers were interviewed about barriers and facilitators to support their loved ones to adhere to a prehabilitation program.

### Data analysis

Data was collected according to the standards for reporting qualitative research checklist (O'Brien et al. 2014). All interviews were recorded and transcribed verbatim. These transcripts were fragmented and open coded in ATLAS.ti version 9 (ATLAS.ti Scientific Software Development GmbH) (N. V. 2020; Soratto et al. 2020). The open codes were divided into subthemes and themes using thematic analysis (Soratto et al. 2020). The first three interviews of each study population were independently fragmented, coded, and thematized by two researchers (MV and EB). Themes were discussed until consensus was reached. These themes were used as a base for coding the other transcripts.

## Results

### Recruitment and sampling

A total of 45 interviews were conducted with twelve healthcare professionals, seventeen patients, and sixteen informal caregivers. Healthcare professionals involved in the treatment process of patients with NSCLC were two rehabilitation physicians, two pulmonologists, one surgeon, one psychologist, two dieticians, two physical therapists, and two case managers (Table 3). The median age of the healthcare professionals was 44 (range 24–64) years. Most healthcare professionals had more than five years of experience in the treatment of lung cancer, but some healthcare professionals mainly treated patients in general pulmonary rehabilitation. All patients and informal caregivers were interviewed at their homes. The median age of the patients was 65 (range 51–85) years, the median time between diagnosis and surgery was six (range 1–24) weeks, and the median length of hospital stay was four (range 2–11) days. Postoperative complications occurred in 69% of the interviewed patients, of which 64% were Clavien-Dindo grade I, 18% were Clavien-Dindo grade II, and 18% were Clavien-Dindo grade IIIa complications. Median age of the informal caregivers was 62 (range 21–84) years. The relationships of the informal caregivers with the patient were spouse (94%) or son (6%). All participant characteristics are summarized in Table 2. Final themes concerning prehabilitation were described and summarized on a code tree (Table 3) The themes from the interviews are summarized in the text below and in Table 4.

**Table 2:** Characteristics of participating healthcare professionals, patients, and informal caregivers

Parameters^a^	Healthcare professionals (*n* = 12)	Patients (*n* = 17)	Informal caregivers (*n* = 16)
Sex *n* (%)			
Male	3 (25%)	9 (56%)	7 (47%)
Female	9 (75%)	7 (44%)	8 (53%)
Age *n* (%)	44 (24–63)	65 (51–85)	62 (21–84)
21–30 years	1 (8%)	–	1 (6)
31–40 years	3 (25%)	–	–
41–50 years	4 (33%)	1 (6%)	–
51–60 years	3 (25%)	5 (29%)	1 (6%)
61–70 years	1 (8%)	8 (47%)	8 (50%)
71–80 years	–	2 (12%)	5 (31%)
> 80 years	–	1 (6%)	1 (6%)
Interview duration in minutes (range)	25 (24–50)	34 (22–57)	23 (7–35)
BMI in kg/m^2^ (range)	–	25 (21–36)	–
FEV_1_ as % of predicted (range)	–	78 (41–131)	–
DLCO as % of predicted (range)	–	75 (42–110)	–
Smoking *n* (%)			
Current	–	4 (24%)	2 (12%)
Former	–	13 (76%)	7 (44%)
Non-smoker	–	0 (0%)	7 (44%)
Work *n* (%)			
Employed when diagnosed	–	7 (47%)	7 (44%)
Retired	–	10 (59%)	6 (37%)
Not employed	–	0 (0%)	3 (19%)
Weeks between diagnosis and surgery (range)	–	6 (1–24)	–
Length of hospital stay in days (range)	–	4 (2–11)	–
Type of surgery *n* (%)			
Lobectomy	–	12 (70%)	–
Pneumonectomy	–	3 (18%)	–
Wedge resection	–	2 (12%)	–
Neoadjuvant chemotherapy	–	1 (6%)	–
Clavien-Dindo classification n (%)			
0–1	–	12 (71%)	–
II	–	3 (18%)	–
III	–	1 (6%)	–
IV	–	1 (6%)	–
Charlson comorbidity index *n* (%)			
0–3	–	2 (12%)	–
≥ 4	–	15 (88%)	–
Relation with patient *n* (%)			
Spouse	–	–	15 (94%)
Son	–	–	1 (6%)
Function *n* (%)			
Rehabilitation physician	2 (17%)	–	–
Pulmonologist	2 (17%)	–	–
Surgeon	1 (8%)	–	–
Psychologist	1 (8%)	–	–
Dietician	2 (17%)	–	–
Physical therapist	2 (17%)	–	–
Case manager	2 (17%)	–	–

^a^Age, interview duration, BMI, FEV_1_, DLCO, weeks between diagnosis and surgery, and length of hospital stay are presented as median (range)

*BMI* body mass index, *FEV*_*1*_ forced expiratory volume in one second, *DLCO* carbon monoxide lung diffusion capacity

**Table 3:** Code tree

Open codes	Subthemes	Themes
Smoking-cessation	Multidisciplinary interventions	Content of prehabilitation and referral
Focus on postoperative period		
Improving aerobic fitness		
Group or individual physical exercise training		
Improving nutritional status		
Coaching/guidance		
Involved disciplines		
Customized prehabilitation components on indication	Referral	
Screening		
Role case manager		
Better prepared for surgery		
Who benefits?	Reasons to refer	
Inclusion		
Decrease postoperative complications (risk)		
Improving postoperative recovery		
Improving survival		
Short period between diagnosis and surgery	Delay surgery for prehabilitation	Organizational factors
Delay surgery not preferred		
Delay surgery is possible		
Prehabilitation so that surgery is possible		
Planning of appointments	Planning	
Quick referral for multimodal prehabilitation		
Schedule		
Multidisciplinary collaboration	Communication	
Communication with patients		
Developing an application		
Knowledge of healthcare professionals		
Knowledge of patients and informal caregiver		
Digital support		
Involving caregivers in prehabilitation		
Home-based physical exercise training	Location	
Hospital-based physical exercise training		
Physical therapy practice		
Primary care		
Lifestyle clinic		
Patient specific prehabilitation program		
Distance		
Self-confidence	Mental and physical status	Personal factors for participation
Physical fitness		
Stressful period for patients and informal caregivers		
Accepting help		
Capable of surgery		
Concerns about their health and surgery		
Status before surgery		
Willingness to participate	Intrinsic motivational	
Awareness		
Motivation		
Self-management		
Self-discipline		
Preparation for surgery		
Financial barrier	External factors	Environmental factors
Financial facilitator		
Time		
Cultural differences		
Concerns of caregiver	Social support	
Support of caregiver		
Understanding social environment

**Table 4:** Summary of main results of expectations, barriers, and facilitators for prehabilitation before lung cancer surgery for each group of stakeholders

Healthcare professionals	Patients	Informal caregivers
Beliefs It is positive to include all patients, but especially patients with a poor preoperative physical fitness and/or nutritional status Prehabilitation is cost-saving when length of hospital stay decreases Multiple professionals within and between primary and secondary care should be involved in organizing prehabilitation Prehabilitation can best take place in a hospital because of short lines between involved healthcare professionals A longer time period before surgery is possible Facilitators Clear reasons for referral: a decrease in the risk of postoperative complications, improve postoperative recovery and survival Providing information to patients on how they can positively influence their health and functioning Accessible information about prehabilitation in different languages The case manager screens, refers, and coordinates Need for agreements for collaboration with involved healthcare professionals in primary and secondary care Making weekly timeslots available for practitioners to be able to schedule patients quickly for prehabilitation Quick referral, start within one week after diagnosis Pulmonologist/surgeon discusses the possibility about a longer time period before surgery with the patient Adequate skills and knowledge about prehabilitation in primary care is essential for feasibility and effectiveness? Involving informal caregivers should be involved to motivate and help patients to adhere Barriers There are no cut-off values to identify patients who benefit most from prehabilitation The short time period between diagnosis and surgery Prehabilitation is not yet reimbursed by healthcare insurance companies	Beliefs Consider themselves already sufficiently fit for surgery, having a healthy and varied diet Prehabilitation makes no difference in the risk of postoperative complications A better preoperative physical fitness and nutritional status facilitate postoperative recovery Positive attitude to participate in a preoperative physical exercise training program if this was recommended by a physician No need for nutritional support Many hospital appointments should be no barrier for prehabilitation Facilitators Receiving information about preparing for surgery and the surgical procedure itself, and about a healthy lifestyle before surgery Being capable to perform physical exercise training such as endurance training (e.g., walking, cycling, swimming) and resistance training Guidance during the prehabilitation program by a physical therapist Face-to-face contact with a physical therapist, contact with a dietician by phone or video consultation Being capable to make time to perform physical exercise training one to three times weekly Short lines of communication between the patient and healthcare professional during prehabilitation Prehabilitation organized in their own living context Physical exercise training in a group or having an experienced training buddy Support of their loved ones Motivation to do something in preparation for surgery, such as being more physically active to improve their health status In order to deal with their perceived level of stress, some patients prefer counseling by a psychologist Education during the prehabilitation program about the advice/expectations during the postoperative period Barriers Interference by many visits of friends and family due to their cancer diagnosis Prehabilitation organized far from home Unsupervised physical exercise training at home Negative beliefs about a longer waiting time for surgery An increase in the perceived level of stress (makes it especially hard to quit smoking)	Beliefs Consider their loved ones to be adequately fit for surgery Smoking-cessation is more successful when initiated by the loved one They are accessible to the patient to talk about worries and stress Facilitators Supporting smoking cessation of their loved one Prehabilitation in the patient’s living context or in primary care to provide optimal support Capable to support their loved one when prehabilitation has to take place: doing physical exercise training together, trying to motivate, logistical support Good physical condition of their loved one in order to perform physical exercise training together A positive attitude of themselves and willing to change their diet Having the opportunity to make extra time to support their loved one Barriers Perceived level of stress/anxiety to lose their loved one in case of a longer time period before surgery

### Healthcare professionals

Healthcare professionals mentioned a need for consensus on a cut-off value for including or excluding patients with lung cancer for prehabilitation, as well as regarding the cost-effectiveness of prehabilitation. Healthcare professionals preferred that case managers screen patients on preoperative modifiable risk factors. They expected that prehabilitation would be most effective in patients with a poor preoperative physical fitness and/or a poor nutritional status.

*“I would prefer that a case manager screens the patient, connecting patients with healthcare professionals, designing treatment plans, and making sure it all gets done on time.” Healthcare professional 3*

According to all healthcare professionals, patients should be informed on how they can positively influence their health and functioning preoperatively. They mentioned the importance of providing similar and unambiguous information about a healthy lifestyle before surgery to patients, taking into account different cultures, beliefs, or language barriers. Healthcare professionals mentioned the short period between diagnosis and surgery as a barrier for prehabilitation, which might be too short to initiate an effective prehabilitation program. Nevertheless, most healthcare professionals reported that surgery could be delayed safely when the pulmonologist or surgeon decides that a delay of surgery is possible. They mentioned that referring patients to prehabilitation might be facilitated when multiple professionals within and between primary and secondary healthcare are involved in prehabilitation and have weekly time slots available to schedule patients quickly. Most healthcare professionals prefer prehabilitation to take place at the hospital because communication between healthcare professionals within the hospital is easier. Healthcare professionals share an electronic patient file which gives the opportunity for quick referrals to prehabilitation. Furthermore, they expressed more trust in the knowledge of physical therapists working in their own hospital.

*“I have doubts about the knowledge and skills regarding prehabilitation by physical therapists in primary care. So, my preference is to offer prehabilitation in the hospital.” Healthcare professional 3*

Healthcare professionals encouraged informal caregivers to be involved in prehabilitation as they can stimulate their loved ones and know how to motivate them. A barrier is that, currently, patients must pay the costs associated with preoperative preventive interventions themselves. Prehabilitation is not yet reimbursed by Dutch healthcare insurance companies.

*“If patients become more fit preoperatively due to prehabilitation, there will be a faster recovery after surgery and therefore a shorter length of hospital stay. While prehabilitation is not yet standard care and is not reimbursed by the by Dutch healthcare insurance companies, I think this is very effective in lowering healthcare costs.” Healthcare professional 9*

### Patients

Most patients considered themselves sufficiently fit for surgery; they reported a healthy diet and felt no need for a prehabilitation program.

*“My condition was sufficient because I cycled to work every day before the diagnosis. In addition, during the test (cardiopulmonary exercise test) before surgery, I did not have to make an effort to reach the level required to be operated on.” Patient 2*

*“I didn't need to get fit before surgery. I think the pulmonologist thought I was fit enough, because in that lung test (forced expiratory volume in one second) it turned out that I could miss a lung.” Patient 6*

Patients did not know that a better preoperative physical fitness and nutritional status reduces the risk of postoperative complications. When the pulmonologist concluded that their physical fitness, according to the preoperative lung function tests, was sufficient to undergo surgery, patients indicated that they felt no need to prepare for surgery. When the patients received information about prehabilitation, they believed it facilitated postoperative recovery and mentioned that they would participate in prehabilitation if their physician recommended it. Patients said they would prefer to receive information about preparation for surgery, the surgery itself, and a healthy lifestyle before surgery and during the postoperative period.

*“During the consultation at which I was diagnosed with lung cancer, the pulmonologist said that I needed to see a physical therapist to increase my endurance capacity. I was fine with it, but I was never referred and never heard from it again.” Patient 3*

*“The doctor asked what activities I did on a regular day. I replied: all household activities, cleaning, and grocery shopping. Hereafter she concluded: well, in that case preoperative physical exercise training is not necessary.” Patient 8*

Patients indicated that they want to know what physical exercises would be practical for them and that an expert, such as a physical therapist or sports instructor, is the one to decide. A preoperative physical exercise training program, such as endurance training (e.g., walking, cycling, swimming) and resistance training, was most frequently mentioned as an intervention that patients would prefer. In contrast, patients felt no need for nutritional support from a dietician.

*“A physical therapist is the best person to advise me what kind of exercises I should do; after all, he has learned for it.” Patient 1*

Patients reported that they would prefer face-to-face guidance of a physical therapist or personal trainer to improve their preoperative physical fitness. Guidance of a dietician could be done by phone or via video-consulting. If a physician recommended prehabilitation, all patients reported that they felt capable to execute a physical exercise training session one to three times weekly and would change their diet when necessary. Most patients had enough time to participate and otherwise would have made time for prehabilitation. Patients indicated that they had many hospital appointments and medical examinations but that was not seen as a barrier to take part in a prehabilitation program. Some patients stated that they had a busy preoperative period because friends and family visited them at home, which was experienced as a barrier to take part in prehabilitation.

*“Normally I walked every day, but in the last week before the operation I had so many visitors that I lost my walking rhythm, and I could not do anything about my fitness anymore.” Patient 3*

The opportunity of having a direct communication with a healthcare professional was mentioned as a facilitator for patients to take part in a prehabilitation program. Patients preferred a physical exercise training program organized in primary care, because it fits better within their living context. Group-based exercises or having a training buddy were preferred by most patients, because of contact with other patients and motivational reasons. A long travel distance was seen as a barrier to participate in prehabilitation.

*“I would have liked to do physical exercises in a group, because it allows you to talk to other patients and it helps to stay motivated.” Patient 9*

Unsupervised prehabilitation at home was seen as a barrier for most patients, as they mentioned a lack of self-discipline. Patients felt they needed the support of their loved ones and preferred that they were able to join the appointments in a prehabilitation program. Some patients described that the intense and stressful period before surgery motivated them to be more physically active and to improve their health. Other patients indicated they were worried about the surgery and the outcomes and felt the need for a preoperative consultation by a psychologist, but this was not offered.

*“I missed psychological support in the entire process. When the doctor said “we found a tumor in your lung”, I really would have liked to have a conversation with a psychologist, because your world is falling apart.” Patient 15*

Patients would like to receive more information about the postoperative period during a prehabilitation program (e.g., what to expect, how to deal with side effects and complications, medication use, process emotions).

*“They write in leaflets what you can and cannot do after surgery in case of fever, regarding medication, et cetera, but not about the preparation for surgery in terms of physical exercise training and nutritional support. I would consider that information about physical exercises is being of additional value.” Patient 4*

*“I would have liked information about physical preparation before surgery. I went to the rehabilitation center where I worked until the diagnosis NSCLC and asked if I can do something to improve my physical fitness before the operation, but they mentioned that they did not know what kind of physical exercises were good and safe.” Patient 15*

Some patients suggested that smoking-cessation before surgery was difficult because of the stressful period before surgery. Furthermore, patients reported that healthcare professionals recommended that they could quit smoking after surgery to avoid an increase in the perceived level of stress before surgery.

*“My wife wanted me to stop smoking right away, but the pulmonologist wanted to wait until after surgery to avoid stress before surgery. After surgery I went to the general practitioner myself and asked for help with quitting smoking.” Patient 5*

Patients did not feel the need for smoking-cessation interventions during prehabilitation. Most patients mentioned that they could talk to their spouses and family about their feelings and felt supported. Some patients would not like a delay of their surgery in favor of prehabilitation; they indicated that they wanted their tumor to be removed as soon as possible, and postponing surgery would increase their anxiety. However, some patients said they would accept a delay of two to four weeks to improve their physical fitness preoperatively.

### Informal caregivers

Most informal caregivers considered their loved ones to be adequately fit for surgery. Informal caregivers said smoking-cessation should be a part of a prehabilitation program merely when initiated by the patient instead of persuading the patient; otherwise, they considered it ineffective. Informal caregivers preferred prehabilitation for their loved ones to be organized in their own living context in primary care in order to be able to provide optimal support. Most informal caregivers indicated they were also willing to participate in a physical exercise training with their loved ones and to offer nutritional support.

*“I think it is important to provide support. You just do that as a partner. It is… we have been together for so long for a reason.” Informal caregiver 4*

Most informal caregivers reported that they wanted the tumor to be resected at the earliest convenience and did not prefer a delay of the surgery in favor of prehabilitation. Most informal caregivers were worried to lose their loved ones due to cancer but tried to remain positive and said they could talk to their loved ones about their feelings and concerns.

## Discussion

The aim of this study was to gain insight into beliefs, facilitators, and barriers of (1) healthcare professionals to refer patients to a prehabilitation program, (2) patients to participate in and adhere to a prehabilitation program, and (3) informal caregivers to support their loved ones in prehabilitation. Healthcare professionals mentioned that the period between diagnosis and surgery might be too short to initiate an effective prehabilitation program. Furthermore, according to healthcare professionals, it is essential to make workable agreements and negotiate with health insurers to include prehabilitation in the basic health insurance.

Patients pointed out that they did not know that prehabilitation would reduce the incidence of postoperative complications; however, they did believe that it would enhance their postoperative recovery. They mentioned that a recommendation from their physician to participate in prehabilitation would facilitate their participation in a prehabilitation program. Furthermore, most patients preferred group-based exercises with supervision of a physical therapist or personal trainer. Informal caregivers said that they would prefer prehabilitation in primary care, in their own living context so they could provide optimal support to their loved ones.

Exercise prehabilitation effectively reduces the occurrence of postoperative complications and reduces length of hospital stay in patients undergoing surgery for NSCLC (Gravier et al. 2021; Granger and Cavalheri 2022). Patients need to be informed about the benefits of improving their health status before surgery, preferably by a physician (Agasi-Idenburg et al. 2020). Priority should be given to facilitate a physician’s involvement in informing patients about the value of physical activity and the need to perform physical exercise training and nutritional support.

The most important barrier for prehabilitation mentioned by healthcare professionals was the short period between diagnosis and surgery. However, previous studies have shown that a two-week prehabilitation program for early-stage NSCLC can already be effective to improve postoperative recovery, as well as that a four-week program can be effective to reduce postoperative complications (Liu et al. 2019; Cavalheri and Granger 2022). Furthermore, a delay in surgery of three to four months after diagnosis has been associated with a decreased survival rate for some types of NSCLC compared to receiving surgery within one month, whereas a delay of one month caused no difference in survival (Mayne et al. 2021). In the current study, thirteen out of seventeen patients had to wait at least 4 weeks for surgery, which means that there had been sufficient time to effectively execute a prehabilitation program.

Healthcare professionals mentioned the need for a consensus on a cut-off value for including or excluding patients in order to select the patients who would benefit the most from prehabilitation. Lower preoperative aerobic fitness has shown to be associated with an increased risk for short-term and long-term postoperative complications in several other surgical populations as well (Moran et al. 2016; West et al. 2014; Moyes et al. 2013; Lee et al. 2018). There are field exercise tests that predict which patients are at high risk for postoperative complications, but unfortunately there is a lack of accurate test-specific cut-off values for these practical tests, heterogeneity in tests, and used outcome measures (Voorn et al. 2020). In an optimal situation there is a possibility of identifying high-risk patients before starting the treatment, after which the physical performance status might be improved by prehabilitation in order to reduce a patient’s risk for complications during and/or after treatment (Licker et al. 2017; Stefanelli et al. 2013).

Most patients considered themselves as adequately fit to undergo surgery and therefore did not need a prehabilitation program. This corresponds with the findings of previous studies amongst patients scheduled for colorectal and gynecological surgery; they also considered themselves fit enough for surgery (Agasi-Idenburg et al. 2020; Zanden et al. 2021). When standard pulmonary function tests raise concerns about resectability, such as the FEV_1_ and carbon monoxide lung diffusion capacity (DLCO), fall below 80% of predicted, a cardiopulmonary exercise test (CPET) is performed for surgical decision-making, by evaluating whether the patient’s preoperative aerobic fitness is adequate for surgery (Vansteenkiste et al. 2014; Herdy et al. 2016). If a physician states that a patient is fit enough to undergo surgery, patients mentioned they did not feel the need to do anything in order to prepare for surgery. Another study found that healthcare professionals usually assume that patients understand the plan of care explained because they did not always ask for the patient's opinion. This is partly because the patient did not always express their opinions themselves, and there was no shared decision-making about treatment (Berger et al. 2017).

### Implementation of prehabilitation in usual care

The results from this study provide valuable information to implement a prehabilitation program before lung surgery that considers the facilitators and barriers of healthcare professionals, patients, and informal caregivers. For developing a feasible prehabilitation program for patients to adhere, it is important that content and context is made as optimal as possible. This study shows that there are many facilitators to set up a feasible prehabilitation program. When prehabilitation becomes usual care, it is important that (1) health professionals know when to refer patients to prehabilitation and that there is a clear application procedure to enroll in a prehabilitation program (2) that patients receive a referral and recommendation for prehabilitation and that patients are adequately informed about the purpose and benefits of prehabilitation (e.g., leaflets, website of the hospital, improving the communication between healthcare professionals and patients). The current study shows that patients were motivated to participate when prehabilitation is recommended by a physician. Knowing the positive effects of prehabilitation before lung resection on postoperative complications, prehabilitation should be considered to become part of usual care.

Patients indicated that they would prefer group training sessions organized in their own living context. A previous study has shown that group-based postoperative physical exercise training for operable lung cancer had social benefits in addition to improved physical fitness in addition to improving exercise adherence, like good social relations with other patients and learning from each other’s experiences (Missel et al. 2019). Thereby, patients prefer a prehabilitation program supervised by a specialized healthcare professional with the possibility that their loved ones could interact as well. Contrary to the patients, most healthcare professionals preferred prehabilitation to take place in the hospital, because communication with other involved healthcare professionals is easier for them. Furthermore, multiple professionals within and between primary and secondary healthcare should be involved in the context of prehabilitation. However, the Dutch government recommends that 50% of care must take place in the living environment of the patient instead of in a healthcare institution by 2030 (Mission document Top sectors and Innovation policy. 2022).

### Strengths and limitations

The present study provides detailed qualitative data on beliefs, preferences, barriers, and facilitators of prehabilitation from the perspective of healthcare professionals, patients with operable NSCLC, and informal caregivers. Ascertaining what is meaningful to patients in the perioperative period in order to participate in a prehabilitation program may be challenging, but is fundamental to clinical patient care (DiGloia et al. 2016), as engagement of patients in their care has been associated with improved clinical outcomes and care experience (Carman et al. 2013). Limitations of the study should also be acknowledged. First, recruitment for healthcare professionals and scheduling interviews was difficult due to hectic periods in pulmonology departments in the medical centers because of the coronavirus disease 2019 pandemic. As such, not all healthcare professionals had extensive experience in the treatment of patients with lung cancer, but they did have experience in treatment of patients with other lung diseases. Second, both pulmonologists interviewed for this study were employed in the same medical center, with the same organization of care and information provision. This might have resulted in reduced richness of the data. Third, patients who participated in this study had not been offered to participate in a prehabilitation program. This means that future studies are needed to evaluate experiences with a prehabilitation program that can be developed with the results from the current study.

In order to facilitate healthcare professionals to refer patients to a prehabilitation program directly after the diagnosis of NSCLC, agreements about the preoperative screening, assessment, and enrollment in prehabilitation is needed. With sufficient time between diagnosis and surgery, prehabilitation could be organized in primary care and it is therefore essential to make workable agreements between healthcare professionals and negotiate with health insurance companies to reimburse prehabilitation. Furthermore, it is recommended to focus on the inclusion of high-risk patients with NSCLC prehabilitation as part of usual care because of the positive effects of prehabilitation on surgical outcomes (Beck et al. 2020).

## Conclusion

In order to be able to start a prehabilitation program as soon as possible after the diagnosis of lung cancer, agreement of preoperative screening and assessment is needed to ensure adequate patient selection, and multidisciplinary collaboration of healthcare professionals within primary and secondary care in referring patients to prehabilitation seems vital. The first step is to inform patients about the purpose and effects of prehabilitation in a one-to-one conversation by the pulmonologist and/or case manager. The next step is to consider patient preferences in organizing an individual or group-based program in their own living context under the supervision of a trained physical therapist. Patients report that the support of their loved ones in their own living context is essential for their adherence to a prehabilitation program. Therefore, it would be wise to involve informal caregivers into the program.

## Data Availability

The data underlying this article will be shared on reasonable request to the corresponding author.

## References

[CR1] Agasi-Idenburg CS, Zuilen MK, Westerman MJ, Punt CJA, Aaronson NK, Stuiver MM (2020). "I am busy surviving"—Views about physical exercise in older adults scheduled for colorectal cancer surgery. J Geriatr Oncol.

[CR2] Beck A, Thaysen HV, Soegaard CH, Blaakaer J, Seibaek L. Investigating the experiences, thoughts, and feelings underlying and influencing prehabilitation among cancer patients: a qualitative perspective on the what, when, where, who, and why. Disabil Rehabil. 2020:1–8. 10.1080/09638288.2020.1762770 32400218

[CR3] Berger ZD, Boss EF, Beach MC (2017). Communication behaviors and patient autonomy in hospital care: a qualitative study. Patient Educ Couns.

[CR4] Carman KL, Dardess P, Maurer M, Sofaer S, Adams K, Bechtel C (2013). Patient and family engagement: a framework for understanding the elements and developing interventions and policies. Health Aff (millwood).

[CR5] Cavalheri V, Granger C. Preoperative exercise training for patients with non-small cell lung cancer. Cochrane Database Syst Rev. 2017;6(6):Cd012020. 10.1002/14651858.CD012020.pub2 PMC6481477 28589547

[CR6] Clavien PA, Barkun J, de Oliveira ML, Vauthey JN, Dindo D, Schulick RD (2009). The Clavien-Dindo classification of surgical complications: five-year experience. Ann Surg.

[CR7] DiGloia AM, Clayton SB, Giarrusso MB (2016). "What matters to you?": A pilot project for implementing patient-centered care. Patient Exp J.

[CR8] Dong B, Wang J, Zhu X, Chen Y, Xu Y, Shao K (2019). Comparison of the outcomes of stereotactic body radiotherapy versus surgical treatment for elderly (≥ 70) patients with early-stage non-small cell lung cancer after propensity score matching. Radiat Oncol.

[CR9] Dutch Institute for Clinical Auditing. Jaarrapportage Dutch Lung Cancer Audit (DLCA) 2018. https://dica.nl/jaarrapportage-2018/dlca. Accessed 14 Sept 2022.

[CR10] Granger CL, Irving L, Antippa P, Edbrooke L, Parry SM, Krishnasamy M (2018). CAPACITY: a physical activity self-management program for patients undergoing surgery for lung cancer, a phase I feasibility study. Lung Cancer.

[CR11] Granger C, Cavalheri V. Preoperative exercise training for people with non-small cell lung cancer. Cochrane Database Syst Rev. 2022;9:CD012020. 10.1002/14651858.CD012020.pub3 PMC9519181 36170564

[CR12] Gravier FE, Smondack P, Prieur G, Medrinal C, Combret Y, Muir JF (2021). Effects of exercise training in people with non-small cell lung cancer before lung resection: a systematic review and meta-analysis. Thorax.

[CR13] Herdy AH, Ritt LE, Stein R, Araujo CG, Milani M, Meneghelo RS (2016). Cardiopulmonary exercise test: background, applicability and interpretation. Arq Bras Cardiol.

[CR14] Kozower BD, Sheng S, O'Brien SM, Liptay MJ, Lau CL, Jones DR, et al. STS database risk models: predictors of mortality and major morbidity for lung cancer resection. Ann Thorac Surg. 2010;90(3):875–81; 81–3. 10.1016/j.athoracsur.2010.03.115 20732512

[CR15] Leduc C, Antoni D, Charloux A, Falcoz PE, Quoix E. Comorbidities in the management of patients with lung cancer. Eur Respir J. 2017;49(3). 10.1183/13993003.01721-2016 28356370

[CR16] Lee CHA, Kong JC, Ismail H, Riedel B, Heriot A (2018). Systematic review and meta-analysis of objective assessment of physical fitness in patients undergoing colorectal cancer surgery. Dis Colon Rectum.

[CR17] Licker M, Karenovics W, Diaper J, Frésard I, Triponez F, Ellenberger C (2017). Short-term preoperative high-intensity interval training in patients awaiting lung cancer surgery: a randomized controlled trial. J Thorac Onc.

[CR18] Liu Z, Qiu T, Pei L, Zhang Y, Xu L, Cui Y, et al. Two-Week Multimodal Prehabilitation Program Improves Perioperative Functional Capability in Patients Undergoing Thoracoscopic Lobectomy for Lung Cancer: A Randomized Controlled Trial. Anesth Analg. 2019;840–849–30 10.1213/ANE.0000000000004342 31348053

[CR40] Lung Cancer Statistics. London: World Cancer Research Fund International. https://www.wcrf.org/cancer-trends/lung-cancer-statistics. Accessed 20 Mar 2020

[CR19] Mayne NR, Elser HC, Darling AJ, Raman V, Liou DZ, Colson YL (2021). Estimating the impact of extended delay to surgery for stage I non-small-cell lung cancer on survival. Ann Surg.

[CR20] Michie S, van Stralen MM, West R (2011). The behaviour change wheel: a new method for characterising and designing behaviour change interventions. Implement Sci.

[CR21] Missel M, Borregaard B, Schoenau MN, Sommer MS (2019). A sense of understanding and belonging when life is at stake-Operable lung cancer patients' lived experiences of participation in exercise. Eur J Cancer Care (engl).

[CR23] Moran J, Wilson F, Guinan E, McCormick P, Hussey J, Moriarty J (2016). Role of cardiopulmonary exercise testing as a risk-assessment method in patients undergoing intra-abdominal surgery: a systematic review. Br J Anaesth.

[CR24] Moyes LH, McCaffer CJ, Carter RC, Fullarton GM, Mackay CK, Forshaw MJ (2013). Cardiopulmonary exercise testing as a predictor of complications in oesophagogastric cancer surgery. Ann R Coll Surg Engl.

[CR25] Verhoeven N (2020) Thematische analyse: patronen vinden bij kwalitatief onderzoek. Boom uitgevers, Amsterdam

[CR26] Netherlands Cancer Registry. Incidence of non-small cell lung cancer in the Netherlands according to Type of Histology (1990–2019) (cited 2020): Comprehensive Cancer Center the Netherlands (IKNL); 2016. http://cijfersoverkanker.nl. Accessed 30 Mar 2020.

[CR27] O'Brien BC, Harris IB, Beckman TJ, Reed DA, Cook DA (2014). Standards for reporting qualitative research: a synthesis of recommendations. Acad Med.

[CR28] Sebio Garcia R, Yanez Brage MI, Gimenez Moolhuyzen E, Granger CL, Denehy L (2016). Functional and postoperative outcomes after preoperative exercise training in patients with lung cancer: a systematic review and meta-analysis. Interact Cardiovasc Thorac Surg.

[CR29] Sebio Garcia R, Yanez-Brage MI, Gimenez Moolhuyzen E, Salorio Riobo M, Lista Paz A, Borro Mate JM (2017). Preoperative exercise training prevents functional decline after lung resection surgery: a randomized, single-blind controlled trial. Clin Rehabil.

[CR30] Senan S (2013). Surgery versus stereotactic radiotherapy for patients with early-stage non-small cell lung cancer: more data from observational studies and growing clinical equipoise. Cancer.

[CR31] Shukla A, Granger CL, Wright GM, Edbrooke L, Denehy L (2020). Attitudes and perceptions to prehabilitation in lung cancer. Integr Cancer Ther.

[CR32] Soratto J, Pires DEP, Friese S. Thematic content analysis using ATLAS.ti software: Potentialities for researchs in health. Rev Bras Enferm. 2020;73(3):e20190250. 10.1590/0034-7167-2019-0250 32321144

[CR33] Stefanelli F, Meoli I, Cobuccio R, Curcio C, Amore D, Casazza D (2013). High-intensity training and cardiopulmonary exercise testing in patients with chronic obstructive pulmonary disease and non-small-cell lung cancer undergoing lobectomy. Eur J Cardiothorac Surg.

[CR34] Templeton R, Greenhalgh D (2019). Preoperative rehabilitation for thoracic surgery. Curr Opin Anaesthesiol.

[CR35] Thomas G, Tahir MR, Bongers BC, Kallen VL, Slooter GD, van Meeteren NL (2019). Prehabilitation before major intra-abdominal cancer surgery: a systematic review of randomised controlled trials. Eur J Anaesthesiol.

[CR22] Top sectors and Innovation policy. Mission document. http://www.topsectoren.nl/missiesvoordetoekomst/documenten/kamerstukken/2019/april/29-04-2019/missiedocument. Accessed 15 Apr 2022.

[CR41] van der Zanden V, van der Zaag-Loonen HJ, Paarlberg KM, Meijer WJ, Mourits MJE, van Munster BC. PREsurgery thoughts—thoughts on prehabilitation in oncologic gynecologic surgery, a qualitative template analysis in older adults and their healthcare professionals. Disabil Rehabil. 2021:1–11. 10.1080/09638288.2021.1952319 34283686

[CR36] Vansteenkiste J, Crino L, Dooms C, Douillard JY, Faivre-Finn C, Lim E, et al. 2nd ESMO Consensus Conference on Lung Cancer: early-stage non-small-cell lung cancer consensus on diagnosis, treatment and follow-up. Ann Oncol. 2014;25(8):1462–74. 10.1093/annonc/mdu089 24562446

[CR37] Verstegen NE, Oosterhuis JW, Palma DA, Rodrigues G, Lagerwaard FJ, van der Elst A (2013). Stage I–II non-small-cell lung cancer treated using either stereotactic ablative radiotherapy (SABR) or lobectomy by video-assisted thoracoscopic surgery (VATS): outcomes of a propensity score-matched analysis. Ann Oncol.

[CR38] Voorn MJJ, Franssen RFW, Verlinden J, Bootsma GP, de Ruysscher DK, Bongers BC, et al. Associations between pretreatment physical performance tests and treatment complications in patients with non-small cell lung cancer: a systematic review. Crit Rev Oncol Hematol. 2020:103207. 10.1016/j.critrevonc.2020.103207 33383208

[CR39] West MA, Lythgoe D, Barben CP, Noble L, Kemp GJ, Jack S (2014). Cardiopulmonary exercise variables are associated with postoperative morbidity after major colonic surgery: a prospective blinded observational study. Br J Anaesth.

